# Human and mouse albumin bind their respective neonatal Fc receptors differently

**DOI:** 10.1038/s41598-018-32817-0

**Published:** 2018-10-02

**Authors:** Jeannette Nilsen, Malin Bern, Kine Marita Knudsen Sand, Algirdas Grevys, Bjørn Dalhus, Inger Sandlie, Jan Terje Andersen

**Affiliations:** 1 Centre for Immune Regulation (CIR) and Department of Immunology, Oslo University Hospital Rikshospitalet, Oslo, Norway; 2 Institute of Clinical Medicine and Department of Pharmacology, University of Oslo, Oslo, Norway; 3 Department of Biosciences, University of Oslo, Oslo, Norway; 4 Department of Medical Biochemistry, Oslo University Hospital Rikshospitalet and University of Oslo, Oslo, Norway

## Abstract

Albumin has a serum half-life of three weeks in humans and is utilized to extend the serum persistence of drugs that are genetically fused or conjugated directly to albumin or albumin-binding molecules. Responsible for the long half-life is FcRn that protects albumin from intracellular degradation. An in-depth understanding of how FcRn binds albumin across species is of importance for design and evaluation of albumin-based therapeutics. Albumin consists of three homologous domains where domain I and domain III of human albumin are crucial for binding to human FcRn. Here, we show that swapping of two loops in domain I or the whole domain with the corresponding sequence in mouse albumin results in reduced binding to human FcRn. In contrast, humanizing domain I of mouse albumin improves binding. We reveal that domain I of mouse albumin plays a minor role in the interaction with the mouse and human receptors, as domain III on its own binds with similar affinity as full-length mouse albumin. Further, we show that P573 in domain III of mouse albumin is required for strong receptor binding. Our study highlights distinct differences in structural requirements for the interactions between mouse and human albumin with their respective receptor, which should be taken into consideration in design of albumin-based drugs and evaluation in mouse models.

## Introduction

Albumin consists of 67% α-helices connected via flexible loops that make up three homologous domains (DI, DII and DIII)^1^. Each domain contains hydrophobic pockets that allow albumin to accommodate and transport various small and insoluble molecules, such as fatty acids, hormones, toxins and drugs, throughout the body^2^. Albumin is the most abundant protein in blood with an average serum half-life of three weeks in humans, a feature only shared with immunoglobulin G (IgG) antibodies^3–5^. Although structurally and functionally unrelated, both albumin and IgG possess the ability to bind a cellular receptor, named the neonatal Fc receptor (FcRn), which is broadly expressed and protects against lysosomal degradation^6,7^. Mice lacking expression of the receptor exhibit only about 40% and 25% of normal serum levels of albumin and IgG, respectively, and the half-life of injected albumin and IgG is considerably shorter than in wild-type (WT) mice^8–11^.

FcRn is a major histocompatibility class I-like molecule consisting of a unique transmembrane heavy chain, with three extracellular domains (α1, α2 and α3), which is non-covalently associated with the common soluble β2-microglobulin (β2m)^12–14^. The binding sites for albumin and IgG on FcRn are distinct and non-overlapping, and binding of both ligands is pH-dependent, occurring only at acidic pH below 6.5 and not at neutral pH^11,15–18^. This enables FcRn to rescue albumin and IgG from lysosomal degradation, as binding readily occurs in acidified endosomes and cease at the cell surface when exposed to the physiological pH of the extracellular surroundings. The mechanisms by which FcRn transports IgG intracellularly have been studied in detail. IgG enters the endosomal pathway when taken up by fluid-phase pinocytosis, but instead of following the route to the lysosomes, it may engage FcRn in endosomes followed by sorting to the cell surface at the side of entry or to the opposite side of polarized cells^7,19–22^. Recent publications support that albumin follows the same cellular pathways as IgG^23–25^.

To fully understand albumin biology, it is important to unravel how FcRn binds and transports albumin. Conserved histidine residues in both FcRn (H166) and albumin (H464, H510 and H535) form intramolecular interactions at acidic pH that are crucial for binding^15,16,18,26^. H166 of the α2-domain in the human FcRn (hFcRn) heavy chain stabilizes a loop in the α1-domain and conserved tryptophan residues (W51, W53, W59 and W61) within the loop^16,18,26,27^. This allows binding of W53 and W59 to hydrophobic pockets in DIII of human serum albumin (HSA) that are sustained in open conformations by H464, H510 and H535^18,26^. DIII of HSA contains the main binding site for hFcRn, but while recombinant DIII on its own can bind the receptor, the affinity is much weaker than of full-length HSA^16,28,29^. More recently, we demonstrated that the reduced affinity is due to a contribution from DI in the complete HSA molecule, as targeting selected residues within two surface-exposed loops in DI (loop I: residues 81–89, loop II: 105–114) either decreased or increased binding to hFcRn when mutated to alanine^18,26,28^.

The half-life property of albumin is used to improve therapeutic efficacy of drugs by extending their serum persistence^30,31^, and engineering of HSA for improved FcRn binding is now explored to further optimize dosing frequency^26,32^. Rodents are extensively used to test WT and engineered HSA-drug fusions, but we have revealed that there are great differences in cross-species FcRn-albumin binding that challenges such evaluations. Specifically for mouse and human, mouse FcRn (mFcRn) binds HSA weakly compared to mouse serum albumin (MSA), whereas hFcRn binds MSA more strongly than HSA^32–34^. We have demonstrated that sequence variation in DIII is largely responsible for the cross-species differences, as MSA with human DIII, instead of mouse DIII, showed comparable binding as WT HSA to the mouse and human forms of FcRn, while binding of HSA with mouse DIII to both receptors improved, but showed even stronger binding than WT MSA towards hFcRn^34^.

In the current study, we aimed to investigate how differences in the amino acid composition of DI between MSA and HSA affect receptor binding. We found that targeting residues within the two DI loops of HSA gave rise to either increased or decreased hFcRn binding when mutated to the corresponding mouse residues. In contrast, substitution of residues in the two loop regions of MSA did not affect binding to hFcRn. Furthermore, HSA with mouse DI (mDI), instead of human DI (hDI), showed weaker binding to hFcRn than WT HSA, whereas improved binding was measured for MSA with hDI. Importantly, recombinant DIII of MSA bound to hFcRn and mFcRn with similar affinity as the full-length molecule, supporting that DI of MSA does not contribute much to the interactions. Lastly, we show that P573 of MSA is crucial for strong receptor binding and that the single amino acid difference between HSA (K573) and MSA at this position contributes greatly to distinct cross species binding. Thus, there are considerable differences in the structural requirements for the interactions between HSA and MSA with their respective receptors, and across the two species. Our findings shed new light on albumin biology across species and must be taken into consideration during development and evaluation of albumin-based therapeutics in preclinical mouse models.

## Results

### Species analysis of the FcRn-albumin interaction

**Figure 1:**
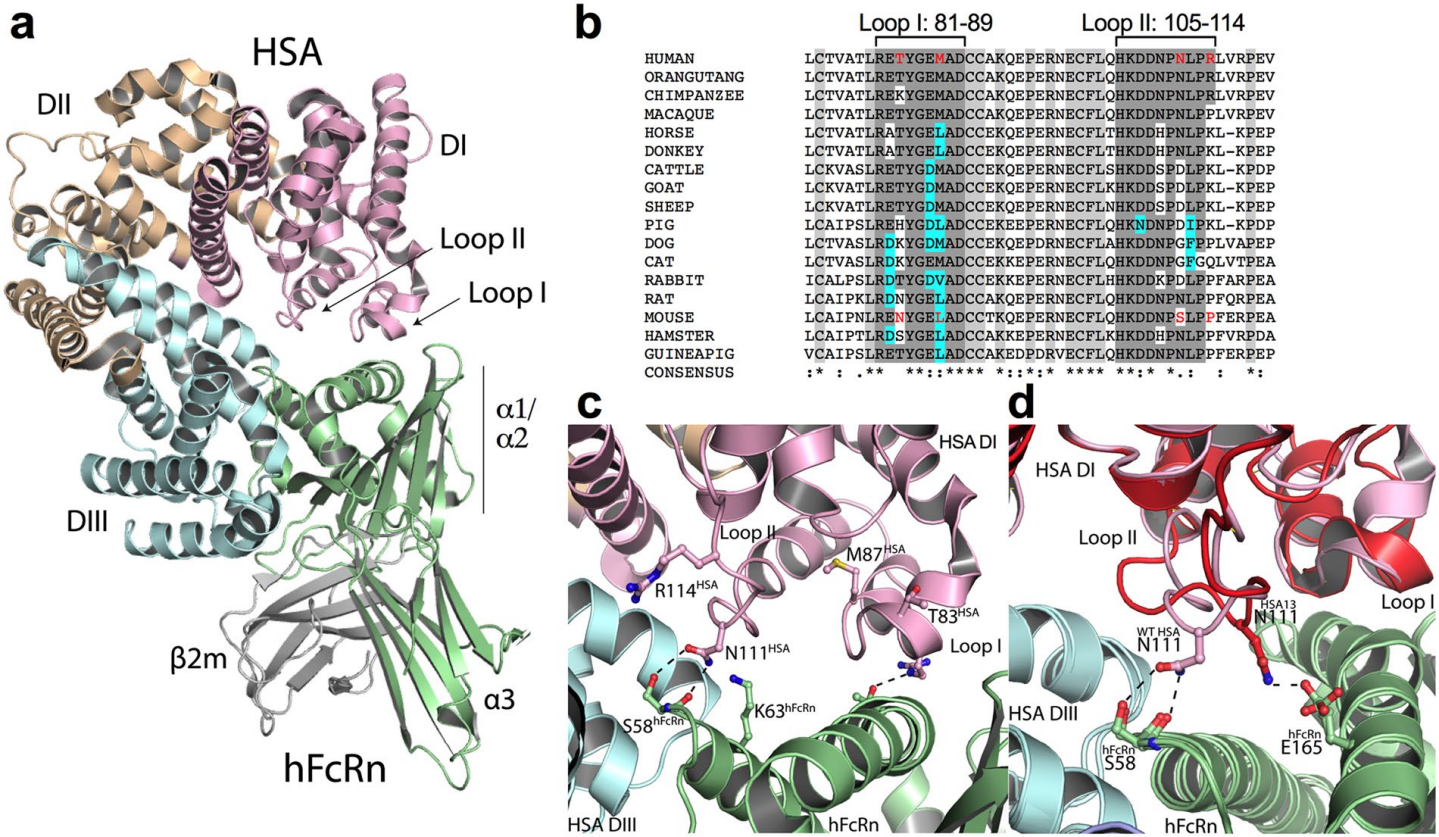
DI and DIII of HSA interact with hFcRn. (**a**) An illustration of the co-crystal structure of WT HSA in complex with hFcRn. DI, DII and DIII of HSA are colored in pink, orange and cyan, respectively, whereas for hFcRn, the heavy chain is shown in green and the β2m subunit in gray. The three domains, α1, α2 and α3, of hFcRn are indicated. (**b**) Alignment of the amino acid residues 74–120 of albumin DI from multiple species. Two DI loops are indicated. Amino acids that are identical to or share the same characteristics as the corresponding human loop residue are highlighted in dark gray and cyan, respectively. The positions in red are the loop residues that differ between HSA and MSA. Conserved amino acids flanking the loops are highlighted in light gray. The alignment was designed using the Clustalω software. (**c**) A close-up of the interface between WT HSA DI and hFcRn, showing interacting residues and amino acids targeted in this study. (**d**) A close-up of a superposition of the WT HSA-hFcRn (*pink*) and HSA13-hFcRn (*red*) co-crystal structures, showing two different conformations of loop II in DI. The figures were made using PyMOL with the crystal structure data of WT HSA-hFcRn and HSA13-hFcRn^18,26^.

Albumin consists of three domains (DI, DII and DIII) connected via flexible loops (Fig. 1a). While DIII is the principal binding domain, two surface-exposed loops in the N-terminal DI of HSA, encompassing residues 80–89 (loop I) and 105–114 (loop II) contribute to optimal hFcRn binding^16,18,26,28^. We have previously uncovered large differences in binding of albumin to FcRn across species, which has consequences when HSA-based therapeutics are evaluated in mouse models^32–34^. The challenge is that HSA binds weakly to mFcRn, while MSA binds more strongly than HSA to hFcRn^32–34^. To gain insight into these differences, we aligned the amino acid sequences of albumin from human and mouse with that of 15 other species (Supplementary Fig. S1), and found that conservation within DI and DIII were 67.1% and 67.9%, respectively, and 68.9% for the non-interacting DII. Regarding mouse and human, the conservation in DI, DII and DIII were 67.6%, 75.6% and 74.0%, respectively. Examining the two exposed loops within DI, we found that seven out of nine amino acids in loop I, and six out of ten in loop II, are either fully conserved or replaced with an amino acid with similar characteristics (Fig. 1b). In loop I, the amino acid in position 83 is the least conserved, and vary from a threonine in humans to a serine, histidine, asparagine and lysine among the other species, suggesting that it may be less important for FcRn binding. Notably, the side chain of T83 does not contact hFcRn or any other residues of HSA in two available co-crystal structures (Fig. 1c), and we previously found that T83 in HSA could be replaced with alanine without affecting hFcRn binding^18,26,28^. Residues 109, 111 and 114 are the most variable in loop II. Interestingly, albumin from human, orangutan and chimpanzee have an arginine in position 114, which is replaced by a lysine in another six of the seventeen species. With the exception of Q114 in cat, albumin from mouse and the remaining six species contain a proline at this position. This amino acid difference may affect the degree of flexibility of the loop, which noted from the HSA-hFcRn co-crystal structures, is able to adopt different conformations (Fig. 1d)^18,26^.

Next, we aligned the amino acid sequence of FcRn from ten species and looked at conservation in the areas involved in binding of DI of HSA (Supplementary Fig. S2). To identify the regions involved in binding, we examined two HSA-hFcRn co-crystal structures^18,26^, and observed that the interface to loop II of DI differed slightly, as the loop had different conformations (Fig. 1d). In the complex with WT HSA, N111 forms two hydrogen bonds with S58 in the α1-domain of hFcRn (Fig. 1d). S58 and flanking residues of the receptor (Q56 to T65) that face the loop are highly conserved among species. In the second co-crystal, the loop extends towards the α2-domain and N111 interacts with E165 (Fig. 1d). This region of the receptor is less conserved, and in all species but primates, E165 (human numbering) is replaced by a glycine. The interface area between hFcRn and loop I of DI is very similar in the two co-crystal complexes (Fig. 1d), and includes the amino acid stretch from N149 to L163 in the α2-domain of the receptor, which is less conserved overall (Supplementary Fig. S2). Thus, the sequence variation here may contribute to the difference between mouse and human FcRn in binding affinity for albumin.

### Loop swapping affects mouse-human cross species binding differently

**Figure 2:**
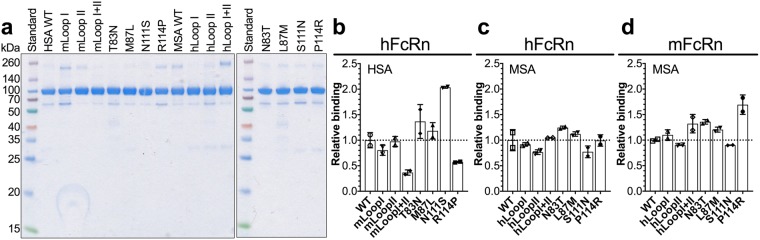
Engineered albumin variants and binding to FcRn. (**a**) 12% SDS-PAGE gel stained with Coomassie Blue showing purified fractions of GST-tagged HSA, MSA and mutant variants. The full-length gels are presented in Supplementary Fig. S9a. (**b**) Relative binding of WT HSA and mutant variants to hFcRn at pH 5.8. Based on EC50 values calculated from ELISA results (Supplementary Fig. S4) where WT HSA was set to 1. (**c,d**) Relative binding of WT MSA and mutant variants to hFcRn (**c**) and mFcRn (**d**) at pH 5.8. Based on EC50 values calculated from ELISA results (Supplementary Fig. S4) where WT MSA was set to 1. The numbers represent the mean ± s.d. of duplicates from one representative experiment.

From the multiple albumin sequence alignment, we found that HSA has four amino acids that differ from MSA in the two DI loops, namely T83N and M87L in loop I and N111S and R114P in loop II (Fig. 1b). To investigate whether the mouse-human differences within the loops affect FcRn binding, we designed a panel of chimeric albumin variants, where loop I, loop II or both were swapped between HSA and MSA (HSA-mLoopI, -mLoopII, -mLoopI + II and MSA-hLoopI, -hLoopII, -hLoopI + II). In addition, we made albumin variants where single amino acid substitutions were introduced (T83N, M87L, N111S, R114P in HSA and N83T, L87M, S111N and P114R in MSA). The mouse-human albumin variants were expressed with a C-terminal GST-tag in HEK293E cells and purified on a GSTrap FF column. None of the mutations affected secretion as they were equally well produced as WT, and purified fractions migrated with expected molecular weights when analyzed by SDS-PAGE and Coomassie staining (Fig. 2a).

To examine the thermal stability of selected variants, we performed a thermal shift assay based on intrinsic fluorescence from aromatic amino acid residues. Tycho NT. 6 was used to measure changes in intrinsic fluorescence at 330 nm and 350 nm with increasing temperature. The fluorescence signals were plotted as a ratio (350 nm/330 nm) and used to calculate the midpoint unfolding inflection temperature (Ti) (Supplementary Fig. S3, Table 1

**Table 1:** Thermal unfolding midpoint temperatures

VARIANT	Ti value^a^ (°C)	VARIANT	Ti value^a^ (°C)
HSA WT	68.9 ± 0.5	MSA WT	67.7 ± 0.3
HSA N111S	69.3 ± 0.1	MSA S111N	67.7 ± 0.1
HSA R114P	69.3 ± 0.1	MSA P114R	67.2 ± 0.3
HSA mLoopI+II	66.7 ± 1.8	MSA hLoopI+II	67.6 ± 0.3
HSA mDI	68.7 ± 0.7	MSA hDI	65.3 ± 0.1
HSA DIII	64.4 ± 1.4	MSA DIII	64.6 ± 0.7
HSA K573P	69.2 ± 0.1	MSA P573K	67.6 ± 0.3

^a^Ti, inflection temperature determined at pH 7.4. The values represent the mean ± s.d. of duplicates.

). A Ti of 68.9 ± 0.5 °C was determined for WT HSA-GST and a similar value of 69.3 ± 0.1 °C was obtained for the two single point mutant variants, N111S and R114P, whereas a slightly lower Ti value of 66.7 ± 1.8 °C was calculated for HSA with both loop I and loop II from MSA. For the WT MSA fusion, a Ti of 67.7 ± 0.3 °C was determined and almost identical values were calculated for MSA with the single point S111N or P114R mutations, as well as for MSA with both loop I and loop II from HSA. Thus, the loop mutations showed no or minor influenced on the thermal stability.

Next, binding of the engineered albumin variants to the mouse and human forms of FcRn was examined at pH 5.8. ELISA revealed that swapping either loop I or loop II in HSA with the corresponding mouse loop did not affect binding to hFcRn, however, introduction of both mouse loops caused reduced binding (Fig. 2b). Testing the single-point HSA mutants of loop I, T83N and M87L, revealed that these substitutions did not influence hFcRn binding, whereas the loop II variants, N111S and R114P, gave rise to enhanced and reduced binding, respectively (Fig. 2b). Weak binding to mFcRn was observed for WT HSA and the mutant variants (Supplementary Fig. S5, a and b). Screening of the MSA variants showed that neither the loop swapping nor the single amino acid mutations had much effect on hFcRn binding, as they bound equally well as WT MSA (Fig. 2c). However, somewhat increased binding to mFcRn was observed for the MSA variant with the single point P114R mutation in loop II (Fig. 2d). None of the albumin variants bound the two receptors at pH 7.4 (Supplementary Fig. S6).

**Figure 3:**
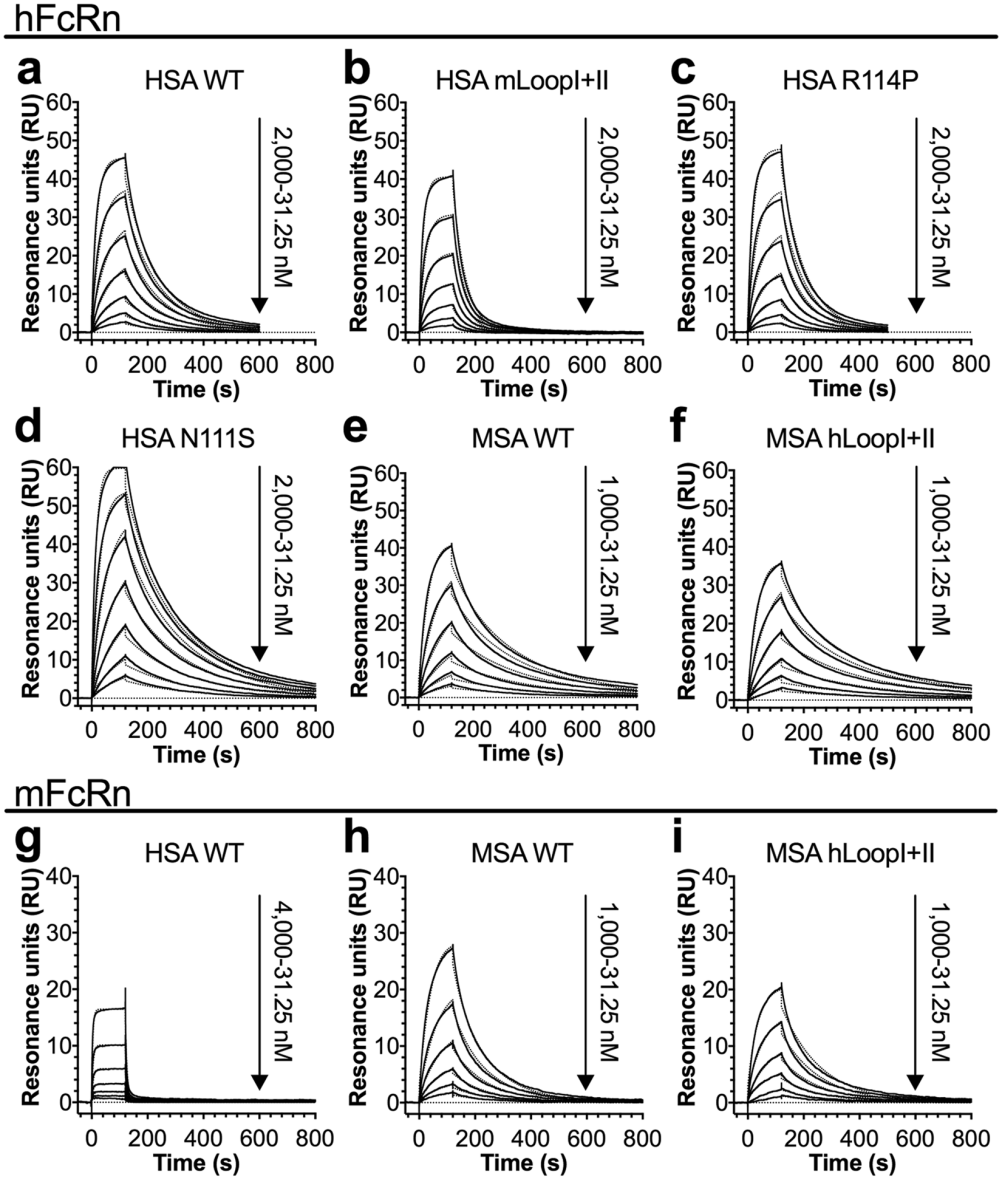
Human-mouse loop swapping of albumin modulates binding to FcRn. (**a–f**) Representative sensorgrams showing binding of titrated amounts of monomeric hFcRn injected over immobilized (200 RU) WT HSA (**a**), HSA mLoopI + II (**b**), N111S (**c**), R114P (**d**), WT MSA (**e**) and MSA hLoopI + II (**f**) at pH 5.5 (—) and the fit of the data to the 1:1 binding model (·····). (**g–i**) Representative sensorgrams showing binding of titrated amounts of monomeric mFcRn injected over immobilized (200 RU) WT HSA (**g**), WT MSA (**h**) and MSA hLoopI + II (**i**) at pH 5.5 (−) and the fit of the data to the 1:1 binding model (·····). Injections were performed with a flow rate of 30 μl/min at 25 °C.

Surface plasmon resonance (SPR) was then used to derive the binding kinetics for selected albumin variants (HSA WT, -mLoopI + II, -N111S, -R114P and MSA WT, -hLoopI + II) by injecting titrated amounts of monomeric receptor over immobilized variants at pH 5.5. Both the human and mouse form of FcRn bound MSA more strongly than HSA (Fig. 3, Table 2). Introduction of the two mouse loops, or the R114P mutation, in HSA modulated the interaction to hFcRn by increasing the dissociation rates, resulting in 2.1- and 1.4-fold weaker binding affinity, respectively (Fig. 3b and c, Table 2). The N111S mutation gave rise to 2.4-fold improved binding due to faster association and slower dissociation (Fig. 3d, Table 2). MSA with two humanized loops bound with close to the same affinity as WT MSA to hFcRn, while 1.5-fold higher affinity was measured towards the mouse receptor (Fig. 3f and i, Table 2). Thus, loop swapping modulated binding of human and mouse albumin to their respective receptors, but did not influence binding of MSA to hFcRn.

**Table 2:** SPR-derived kinetics for binding of albumin variants towards hFcRn and mFcRn.

VARIANT^*a*^	k_a_ (10^4^M^−1^ s^−1^)	k_d_ (10^−3^s^−1^)	K_D_^*b*^ (nM)	χ^2*c*^	Fold Δ^*d*^
**hFcRn**					
HSA WT	2.3 ± 0.2	8.9 ± 0.9	387.0	0.4	1.0
HSA mLoopI + II	2.6 ± 0.1	21.4 ± 0.1	823.1	0.2	+ 2.1
HSA N111S	3.7 ± 0.1	6.0 ± 0.2	162.2	0.7	−2.4
HSA R114P	2.2 ± 0.2	12.2 ± 1.1	554.5	0.4	+ 1.4
HSA mDI	1.4 ± 0.1	20.2 ± 1.4	1442.9	0.2	+ 3.7
HSA DIII	1.1 ± 0.1	34.9 ± 0.7	3172.7	2.1	+ 8.2
HSA K573P	2.7 ± 0.1	0.6 ± 0.1	22.2	0.4	−17.4
MSA WT	3.2 ± 1.0	5.4 ± 1.5	168.8	0.5	1.0
MSA hLoopI + II	5.1 ± 0.1	7.4 ± 0.6	145.1	0.2	−1.2
MSA hDI	6.7 ± 0.6	4.7 ± 0.3	70.2	0.6	−2.4
MSA DIII	4.8 ± 0.1	5.7 ± 0.3	118.8	0.4	−1.4
MSA P573K	2.0 ± 0.1	73.4 ± 3.6	3670.0	0.5	+ 21.7
**mFcRn**					
HSA WT	NA^*e*^	NA	NA	NA	NA
HSA K573P	1.6 ± 0.1	19.7 ± 0.1	1231.3	0.2	NA
MSA WT	3.0 ± 0.1	16.4 ± 2.0	546.7	0.1	1.0
MSA hLoopI + II	2.0 ± 0.1	7.5 ± 0.5	375.0	0.1	−1.5
MSA hDI	2.1 ± 0.1	14.8 ± 1.0	704.8	0.2	+ 1.3
MSA DIII	1.5 ± 0.1	9.3 ± 0.1	620.0	0.2	+ 1.1
MSA P573K	NA	NA	NA	NA	NA

^*a*^The albumin variants were immobilized on CM5 chips and serial dilutions of hFcRn or mFcRn were injected.

^*b*^The kinetic rate constants were obtained using a simple first-order (1:1) Langmuir bimolecular interaction model. The kinetics values represent the mean ± s.d. of duplicates.

^*c*^χ^2^ values resulting from curve fitting using the first-order (1:1) bimolecular interaction model. χ^2^ is a measure of the average squared residual (the difference between the experimental data and the fitted curve).

^*d*^Fold difference in K_D_ compared to the WT interaction.

^*e*^NA, not acquired due to fast kinetics.

### Domain swapping reveals mouse-human binding differences

**Figure 4:**
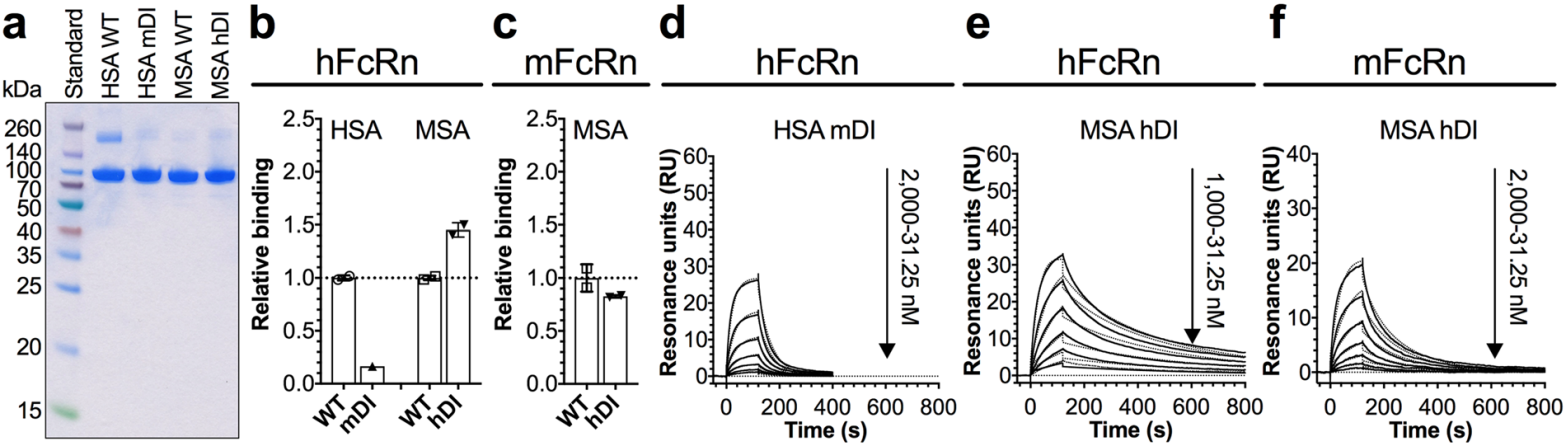
Chimeric DI albumin variants and their FcRn binding properties. (**a**) 12% SDS-PAGE gel stained with Coomassie Blue showing purified fractions of GST-tagged HSA, MSA and chimeric variants. The full-length gel is presented in Supplementary Fig. S9b. (**b,c**) Relative binding of WT and chimeric variants to hFcRn (**b**) and mFcRn (**c**) at pH 5.8, based on EC50 values calculated from ELISA results (Supplementary Fig. S4) where WT was set to 1. The EC50 value of HSA mDI could not be reliably determined, as saturation was not reached. The numbers represent the mean ± s.d. of duplicates from one representative experiment. (**d–f**) Representative sensorgrams showing binding of titrated amounts of monomeric hFcRn (**d–e**) or mFcRn (**f**) injected over immobilized (200 RU) chimeric albumin variants at pH 5.5 (−) and the fit of the data to the 1:1 binding model (·····). Injections were performed with a flow rate of 30 μl/min at 25 °C.

The multiple sequence alignment revealed that DI of HSA and MSA share less sequence identity (67.6%) compared with the other two domains (DII; 75.6%, DIII; 74.0%). To investigate how the sequence variations affect FcRn binding, we produced chimeric albumin variants where the N-terminal DI (residues 1–190) was swapped between HSA and MSA (Fig. 4a). The segment between residue 190 and 210 in HSA intersects DII and DIII at the core of the protein, and thus, to avoid potential mouse-human amino acid mismatch in the domain interfaces that could affect the stability of the chimera, the DI-DII boundary was set to residue 190. The thermal shift assay showed that HSA with mDI had close to the same Ti as WT HSA, whereas 2.4 °C lower Ti was determined for MSA with hDI in comparison to WT MSA (Supplementary Fig. S3, Table 1).

We then measured the effect of swapping on receptor binding using ELISA. At pH 5.8, we detected weaker binding of HSA-mDI than of WT HSA towards hFcRn, whereas MSA-hDI bound more strongly than WT MSA (Fig. 4b). The mouse receptor bound WT HSA and HSA-mDI weakly, and did not discriminate between WT MSA and MSA-hDI (Supplementary Fig. S5, Fig. 4c). None of the chimeric variants bound the two receptors at pH 7.4 (Supplementary Fig. S6). Results from SPR confirmed the weaker binding of HSA-mDI to hFcRn, 3.7-fold compared to WT HSA, and the improved binding by 2.4-fold for MSA-hDI in comparison to WT MSA (Fig. 4d and e, Table 2). MSA-hDI bound with similar affinity as WT MSA towards the mouse receptor (Fig. 4f, Table 2). Thus, at acidic pH, hFcRn preferred hDI over mDI, whereas it did not make a difference for mFcRn.

### DIII binds with similar affinity as full-length MSA

**Figure 5:**
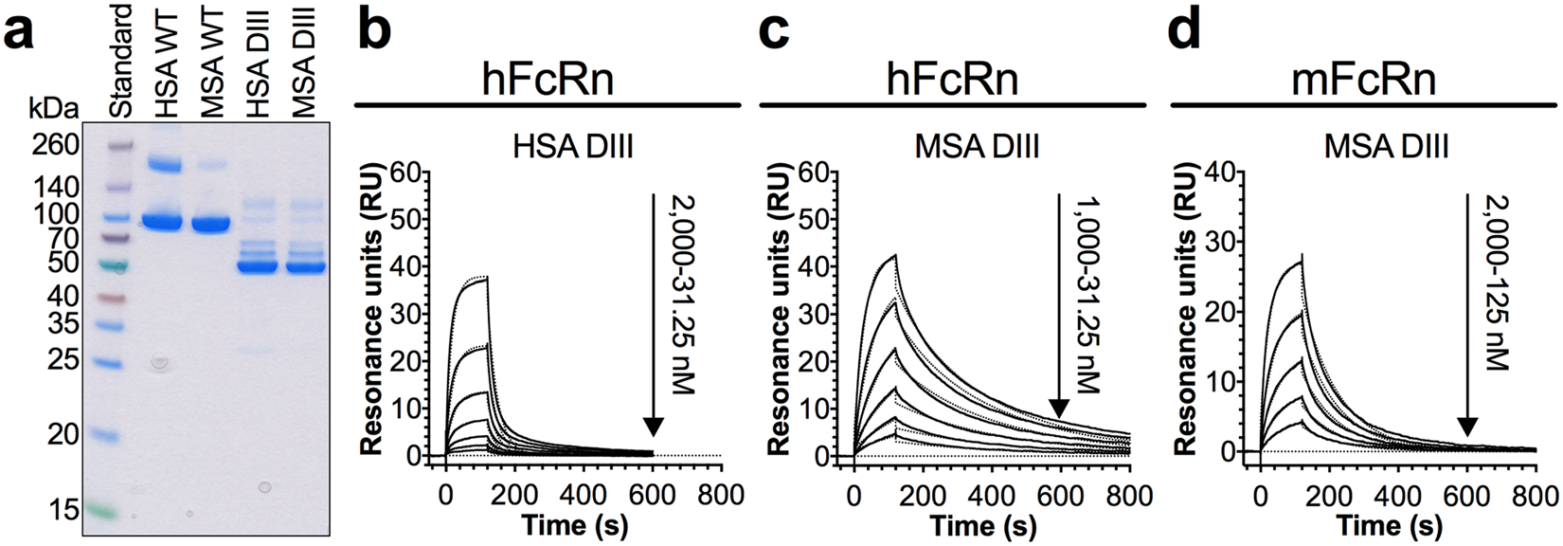
MSA DIII binds more strongly than HSA DIII to FcRn. (**a**) 12% SDS-PAGE gel stained with Coomassie Blue showing purified fractions of GST-tagged DIII and full-length HSA and MSA. The full-length gel is presented in Supplementary Fig. S9c. (**b–d**) Representative sensorgrams showing binding of titrated amounts of monomeric hFcRn (**b,c**) or mFcRn (**d**) injected over immobilized (500 RU) DIII of HSA (**b**) and DIII of MSA (**c**,**d**) at pH 5.5 (−) and the fit of the data to the 1:1 binding model (·····). Injections were performed with a flow rate of 30 μl/min at 25 °C.

Our findings show that the binding of MSA to the human and mouse forms of FcRn was unaffected or improved by humanizing DI. To further examine the role of DI, we expressed recombinant DIII derived from MSA and HSA with a C-terminal GST-tag (Fig. 5a). The thermal shift assay revealed that the DIII fragments had slightly lower Ti values compared to the full-length molecules (Supplementary Fig. S3, Table 1). The binding kinetics were determined using SPR by immobilizing the DIII fragments and injecting titrated amounts of monomeric hFcRn or mFcRn at pH 5.5. In line with previous results^16,28^, binding of HSA DIII to hFcRn was almost 10-fold weaker than that of the full-length molecule (Fig. 5b, Table 2). In stark contrast to the human pair, the binding kinetics of DIII of MSA was only slightly different from that of full-length MSA towards hFcRn (Fig. 5c, Table 2). When tested for binding to mFcRn, 2-fold slower association and almost 2-fold slower dissociation were measured for DIII alone, resulting in similar binding affinity as that measured for full-length MSA (Fig. 5d, Table 2). Our data support that DI of HSA plays a significant role in binding to hFcRn, whereas the contribution from DI of MSA to the interaction is minor in comparison.

### A single amino acid difference in DIII affects cross-species binding

Alignment of albumin sequences shows that human, orangutan and chimpanzee are the only species that express albumin with a lysine in position 573, whereas albumin from mouse and the remaining thirteen species all use proline in this position (Supplementary Fig. S1). We previously showed that replacing the lysine in HSA with a proline had a strong positive effect on binding to both hFcRn and mFcRn and on the circulatory half-life in mice and monkeys^32^.

**Figure 6:**
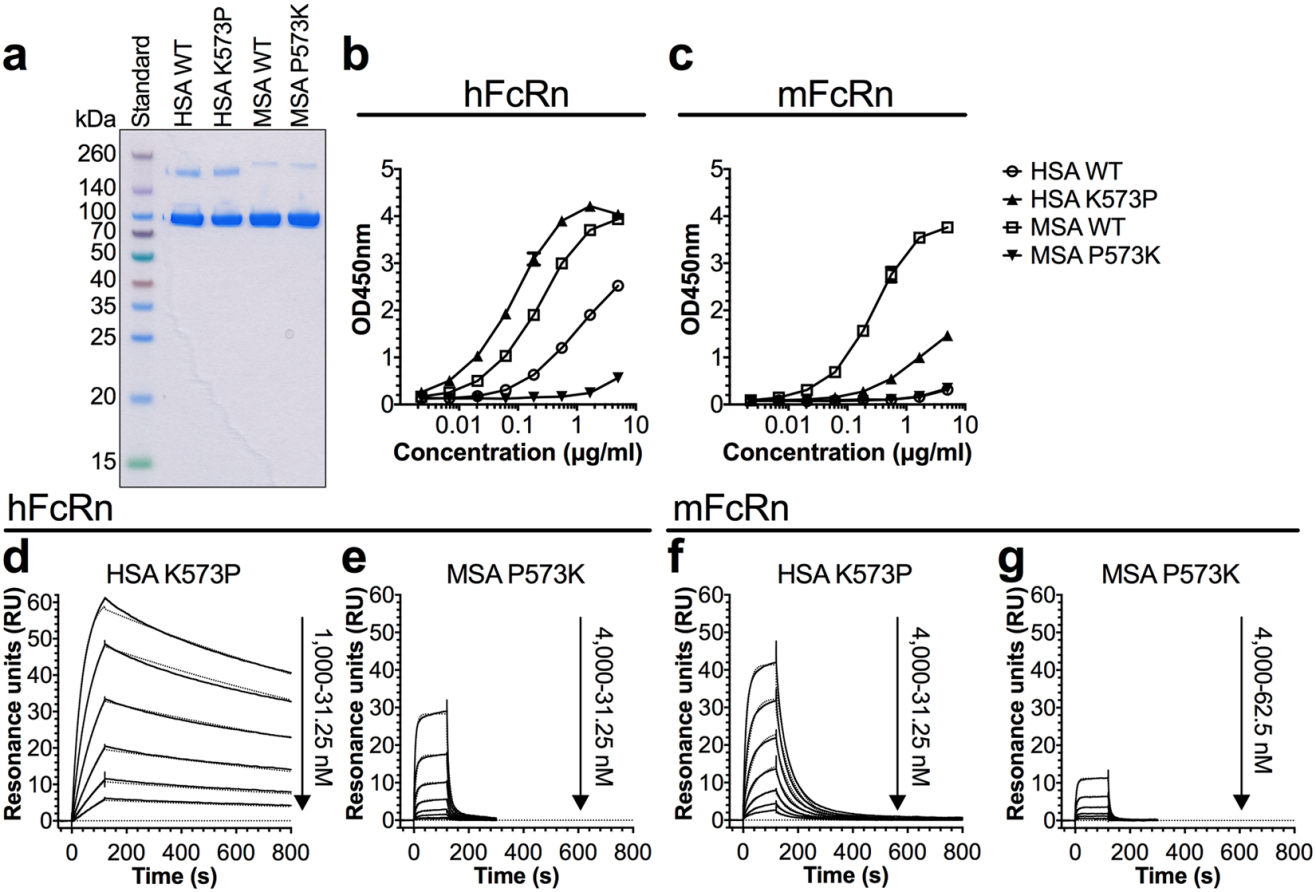
P573 in DIII of albumin promotes binding to FcRn. (**a**) 12% SDS-PAGE gel stained with Coomassie Blue showing purified fractions of GST-tagged HSA, MSA and single point mutant variants. The full-length gel is presented in Supplementary Fig. S9d. (**b-c**) ELISA showing binding of WT and single point mutant variants (HSA K573P and MSA P573K) to hFcRn (**b**) and mFcRn (**c**) at pH 5.8. The numbers represent the mean ± s.d. of duplicates from one representative experiment. (**d–g**) Representative sensorgrams showing binding of titrated amounts of monomeric hFcRn (**d,e**) and mFcRn (**f,g**) injected over immobilized (200 RU) HSA K573P (**d** and **f**) and (500 RU) MSA P573K (**e** and **g**) at pH 5.5 (−) and the fit of the data to the 1:1 binding model (·····). Injections were performed with a flow rate of 30 μl/min at 25 °C.

To further study the importance of this particular amino acid difference in cross-species binding, the proline in MSA was mutated to lysine (P573K) and vice versa in HSA (K573P) (Fig. 6a). The single point mutations did not affect the thermal stability of the proteins (Supplementary Fig. S3, Table 1). Binding to the human and mouse forms of FcRn was measured in ELISA and SPR as before. The K573P mutation in HSA gave rise to 17-fold improved binding to hFcRn, and even stronger binding than that measured for WT MSA (Fig. 6b and d, Table 2). In addition, binding to mFcRn increased considerably, but not to a level beyond that of WT MSA (Fig. 6c and f). In line with these results, introducing the P573K mutation in MSA led to more than 20-fold decreased binding to hFcRn, and to almost 10-fold weaker binding compared with WT HSA (Fig. 6b and e, Table 2). Moreover, binding to mFcRn was also considerably reduced due to the mutation, but the binding kinetics could not be reliably determined (Fig. 6c and g). Thus, the proline of MSA contributes greatly to strong binding to both mFcRn and hFcRn.

## Discussion

In this study, we first examined how sequence variation in DI between mouse and human albumin affects binding to the mouse and human forms of FcRn. In addition to the main binding site for hFcRn in the C-terminal DIII of HSA, we recently discovered that the N-terminal DI, and two-surface exposed loops in particular, contribute to optimal pH-dependent binding to the receptor^16,18,26,28^. Here we identified four amino acid differences between HSA and MSA in the two DI loops. Mutation of all four amino acids in HSA to the corresponding residues in mouse resulted in negative modulation of binding to hFcRn. However, when the mouse residues were introduced individually, it turned out that only one mutation in loop II, R114P, was responsible for reduced hFcRn binding, and that it had actually masked a positive effect of the second loop II mutation, N111S. In the two co-crystal structures of hFcRn in complex with WT HSA or HSA13, loop II of DI have different orientations, which suggests that the loop is a flexible part that can adopt distinct conformations and interactions upon receptor binding^18,26,28^. In the WT HSA-hFcRn complex, N111 forms two favorable contacts with S58 of hFcRn and one unfavorable contact with K63^18^. Notably, substitution of N111 with an alanine also improved binding to hFcRn^28^. Thus, increased receptor binding of mutating N111 to a serine or an alanine, which removes the repulsive interaction and two direct hydrogen bonds, suggests that improved interactions in nearby locations are allowed to occur. Interestingly, albumin from several species expresses an aspartic acid in this position, which could potentially form a favorable salt bridge with K63, and lead to increased binding to hFcRn if introduced in HSA.

R114 does not interact directly with hFcRn, but a proline in this position may affect the flexibility of the loop and explain the decrease in binding to the receptor. R114 is mutated to a glycine in the naturally occurring HSA variant Yahomana-2, and we previously showed that a recombinant form of this variant had almost 2-fold reduced binding affinity for hFcRn compared to WT^28,35^. Loop II extends over a binding pocket, the so-called FA site 1 or drug site 3, located in sub-domain IB of HSA, which also accommodates hemin and bilirubin^36,37^. Interestingly, homozygotes for the Yahomana-2 variant have been shown to bind bilirubin poorly^38^, whereas crystal structures of HSA solved in the presence of ligand have revealed that R114 coordinates binding of hemin to the binding site^36^. Thus, R114 of HSA is not only important for optimal binding to hFcRn, but is also involved in binding of cargo to the binding pocket in DIB. In the multiple sequence alignment, we found that about half of the species included express either a positively charged arginine or lysine in position 114, whereas MSA and the remaining species have a proline. This is interesting in regard to how binding of cargo may affect FcRn binding and transport properties, and subsequent biodistribution, and whether there are differences to such mechanisms between species should be addressed in future studies.

Swapping DI of HSA with mDI led to decreased hFcRn binding, whereas MSA with hDI showed improved binding. This is actually in line with results we previously reported on DIII swapping, where a chimeric variant built from DI-DII of HSA and DIII of MSA was found to bind more strongly than WT MSA to hFcRn^34^. Thus, it is sequence variation in DIII that is mainly responsible for stronger binding of MSA than HSA, whereas hDI is actually favored over mDI in binding to hFcRn. Interestingly, and in contrast to DIII of HSA, DIII of MSA bound with similar binding affinity as full-length MSA to both the human and mouse forms of FcRn, which support that while DI of HSA is crucial for optimal binding to hFcRn, DI of MSA plays only a minor role in binding to the receptors.

Furthermore, we show that a single amino acid residue in position 573 of DIII has great influence on cross species receptor binding^32^. When the amino acid at position 573 of MSA (P573K) and HSA (K573P) was substituted and binding of HSA and MSA could be compared without this amino acid difference, HSA was the strongest binder towards hFcRn (HSA K573P > WT MSA and WT HSA > MSA P573K). The DI contribution from HSA may partly explain the effect. Except for albumin from primates, all other species have P573 in common with MSA. It is therefore tempting to speculate whether the HSA DI contribution evolved to compensate for the P573K mutation, or that the proline was lost after DI got involved, to fine-tune the binding affinity for optimal serum half-life. Moreover, MSA bound more strongly to mFcRn than HSA with the K573P mutation. Only when HSA was equipped with the whole DIII from MSA, did it show as strong binding as MSA^34^. Thus, there must be additional amino acids in MSA DIII that contribute to optimal mFcRn binding.

Receptor binding to the albumin variants was measured at pH 5.5 in PBS containing 0.05% Tween-20. Notably, we observed that the percentage of detergent affected the binding kinetics (Supplementary Fig. S7, Supplementary Table S1). The derived K_D_ for the WT HSA-hFcRn pair was actually 4.3-fold lower in the presence of only 0.005% Tween-20 as compared to 0.05%, whereas binding of WT MSA to the human receptor was 2.5-fold stronger. Interestingly, the WT MSA-mFcRn interaction was affected to a lesser extent as the K_D_ was 1.7-fold lower in the presence of 0.005% Tween-20 compared to 0.05%. Tween-20 contains a fatty acid ester moiety (lauric acid (C12:0)) and a long polyoxyethylene chain. In light of a previous report showing that fatty acids can bind hydrophobic pockets of HSA and modulate hFcRn binding^26^, it may be that Tween-20 can bind albumin and affect receptor binding.

Another interesting question is whether the interactions are modulated through a pH gradient. Interestingly, MSA bound only slightly better than HSA to hFcRn at pH 5.5 (Supplementary Fig. S8). However, while binding of HSA was clearly weakened as the pH increased from pH 5.5 to pH 6.0, binding of MSA was less affected by the pH in this range, which bound more strongly than HSA at both pH 5.8 and 6.0^33,34^. Thus, as binding to FcRn occurs in an endosomal environment that gradually becomes more acidic, MSA may engage the receptor before HSA. Importantly, when engineered HSA and MSA variants were compared with WT at pH 5.5 and 6.0, the mutations showed the same effect on binding to hFcRn as at pH 5.8 (Supplementary Fig. S8).

Our findings should be taken into consideration as they may have implications for development of mouse and human albumin fusions made for pre-clinical testing in conventional and transgenic mice. In addition, drugs may be fused or conjugated to small albumin-binding molecules, such as anti-albumin binding antibody fragments or the albumin-binding domain derived from bacterial *Streptococcal* protein G, which target endogenous albumin following administration^39–42^. To achieve extended drug half-life, it is important that binding to albumin does not negatively influence FcRn binding and transport. Although the binding site on albumin has not been mapped for most albumin-binding molecules, drugs attached to such molecules have acquired prolonged half-life in different animal models^40–42^. In light of our new findings, binding of cross-species reactive albumin-binding drugs may affect the interaction between albumin from mouse and human and FcRn differently. As such, molecules that bind in the DI region may have a greater influence on receptor binding of HSA than of MSA. Thus, to avoid influencing FcRn binding, it may be favorable to target DII of albumin when designing novel albumin-binding molecules.

Fusion to DIII of albumin was previously suggested as an alternative scaffold to alter the serum half-life of coupled molecules^43^. When an anti-carcinoembryonic antigen diabody was fused to DIII of HSA, it resulted in increased blood persistence in mice^43^. However, this was most likely due to the increase in size above the clearance threshold of the kidneys and not dependent on FcRn, as mFcRn binds HSA very weakly. Fusion of diagnostic and therapeutic molecules to full-length albumin may in some cases affect their potency, and the use of the smaller DIII of albumin could then be beneficial. A smaller scaffold may also be preferred when the fusion is large and target accessibility is an issue. Importantly, as mFcRn binding of MSA is less dependent on DI than hFcRn binding of HSA, construction of a MSA DIII-fusion for pre-clinical testing in mice may not reflect the *in vivo* behavior of the corresponding HSA DIII-fusion. Furthermore, to obtain long *in vivo* half-life in the presence of high levels of endogenous albumin, engineering of HSA DIII, which makes it more MSA DIII-like or even a better FcRn binder, would be necessary to increase its ability to compete efficiently with full-length albumin for binding to the receptor.

## Methods

### Cell culture

High Five cells (Invitrogen) were grown in Express FIVE SEF medium (Invitrogen) supplemented with 18 mM L-glutamine and 1% antibiotic-antimyotic (Invitrogen). HEK293E cells (ATCC) were cultured in RPMI 1640 (Sigma-Aldrich) supplemented with 10% heat-inactivated FCS (Sigma-Aldrich), 25 μg/ml streptomycin and 25 U/ml penicillin (Bio Whittaker). Both cell lines tested negative for mycoplasma contamination (MycoAlert^TM^ PLUS Mycoplasma detection kit, Lonza).

### Production of recombinant human and mouse FcRn

Production of His-tagged soluble mouse and human forms of FcRn was performed using a Baculovirus expression vector system, as described previously^44,45^. Viral stocks encoding the receptors were a kind gift from Dr. Sally Ward (University of Texas, Southwestern Medical Center, Dallas, US). Secreted receptor was purified from supernatant using a 5 ml HisTrap HP column supplied with Ni^2+^ ions (GE Healthcare). The column was pre-equilibrated with 1xPBS supplemented with 0.05% sodium azide. The supernatant was adjusted to pH 7.2 by adding 1xPBS/0.05% sodium azide (pH 10.9), before it was applied at a flow rate of 5 ml/min. The column was subsequently washed with 200 ml of 1xPBS, followed by 50 ml of 25 mM imidazole/1xPBS (pH 7.3). Bound receptor was eluted with 250 mM imidazole/1xPBS (pH 7.4) and then up-concentrated and buffer-exchanged to 1xPBS/0.05% sodium azide using Amicon Ultra-15 10 K columns (Millipore). The monomeric fractions were isolated using a HiLoad 26/600 Superdex 200 prep grade column (GE Healthcare) and then up-concentrated using Amicon Ultra-15 10 K columns (Millipore).

### Construction and production of albumin variants

A pcDNA3 vector encoding cDNA of full-length human albumin (585 aa.) in-frame with a C-terminal GST from *Shistosoma Japonicum* has previoulsy been described^29^. The vector was used for sub-cloning of cDNA fragments (Genscript) encoding full-length mouse albumin (584 aa.), DIII variants of HSA (residues 381–585) and MSA (residues 381–584)^16^ or chimeric albumin variants where DI (residues 1–190) derived from HSA and MSA were swapped between the two (HSA-mDI and MSA-hDI). In addition, single point or combinations of mutations were introduced in the full-length sequences of HSA (T83N, M87L, N111S, R114P, K573P, T83N/M87L, N111S/R114P and T83N/M87L/N111S/R114P) and MSA (N83T, L87M, S111N, P114R, P573K, N83T/L87M, S111N/P114R and N83T/L87M/S111N/P114R). The vectors were transiently transfected into HEK293E cells using Polyethylenimine Max (Polysciences) and secreted proteins were purified from harvested supernatant using a GSTrap FF column, as described before^29^. Eluted fractions were up-concentrated and buffer-exchanged to 1xPBS/0.05% sodium azide using Amicon Ultra-15 10 K columns (Millipore). Protein concentrations were measured using a DS-11 spectrophotometer (DeNovix Inc.) and 2 μg of each protein product was analyzed on a 12% NuPAGE SDS-PAGE (Thermo Fisher Scientific).

### Thermal shift assay

The thermal shift assay was performed using Tycho NT. 6 (NanoTemper Technologies). Albumin variants were diluted (0.75 mg/ml) in PBS (pH 7.4) and run in duplicates in capillary tubes. Intrinsic fluorescence was recorded at 330 nm and 350 nm while heating the sample from 35–95 °C at a rate of 3 °C/min. The ratio of fluorescence (350/330 nm) and the Ti were calculated by Tycho NT. 6.

### ELISA

Ninety-six-well ELISA plates (Costar) were coated with a human IgG1 mutant (M252Y/S254T/T256E/H433K/N434F) with specificity for 4-hydroxy-3-iodo-5-nitrophenylacetic acid^46^ (10 µg/ml) in 100 µl PBS (pH 7.4) by incubating over night at 4 °C. The wells were blocked with 250 µl PBS, 4% skimmed milk (PBS/M) for 1 h at RT, and then washed three times with 250 µl PBS, 0.05% Tween 20 (PBS/T) (pH 5.5). His-tagged mFcRn or hFcRn (10 µg/ml) in 100 µl PBS/T/M (pH 5.8) was added per well and incubated for 1 h at RT, before repeating the washing step above. Serial dilutions of albumin variants (5–0.002 µg/ml) were prepared in PBS/T/M (pH 5.8) in duplicates, and 100 µl was added per well and incubated for 1 h at RT. The wells were washed as before. HRP-conjugated anti-GST antibody from goat (GE Healthcare) (1:3000) in 100 μl PBS/T/M (pH 5.8) was added per well and incubated for 1 h at RT. The wells were washed three times with 250 µl PBS/T, before 100 µl tetramethylbenzidine substrate (Calbiochem) and then 100 µl 1 M HCl were added. The absorbance was measured at 450 nm using the Sunrise spectrophotometer (TECAN). The binding responses were fitted and EC50 values extracted using nonlinear regression analysis (log[agonist] vs. response (4 parameter)). The assay was also performed at pH 5.5, pH 6.0 and pH 7.4 and the wash buffer (PBS/T) and dilution buffer (PBS/T/M) were pH-adjusted accordingly.

### SPR

SPR was performed using Biacore T200 (GE Healthcare). Following the manufacturer’s protocol, albumin variants (4–10 μg/ml in 10 mM sodium acetate pH 4.5) were immobilized on CM5 Series S sensor chips using amine coupling chemistry to ~200 RU or 500 RU. Unreacted moieties were blocked with 1 M ethanolamine. Phosphate buffer (177 mM phosphate, 85 mM NaCl, 0.05% Tween 20) at pH 5.5 and phosphate buffer (195 mM phosphate, 85 mM NaCl, 0.05% Tween20) at pH 7.4 were used as running buffer and regeneration buffer, respectively. Kinetic measurements were performed by injecting serial dilutions of soluble monomeric His-tagged hFcRn or mFcRn in the pH 5.5 buffer at a flow rate of 30 μl/min at 25 °C. All binding curves were zero adjusted and the reference flow cell value was subtracted. The binding kinetics were determined using the 1:1 Langmuir binding model provided by the Biacore T200 Evaluation Software, version 3.0.

### Sequence and structural analysis

The amino acid sequences of albumin and FcRn were obtained from the National Center for Biotechnology Information. The albumin sequences of human (AAA98797), orangutan (NP_001127106.2), chimpanzee (XP_517233.3), macaque (NP_001182578.1), horse (NP_001075972.1), donkey (AAV28861), cattle (AAA51411), goat (ACF10391), sheep (NP_001009376), pig (AAA30988.1), dog (CAB64867.1), cat (CAA59279.1), rabbit (NP_001075813), hamster (ABR68005.1), guinea pig (AAQ20088.1), rat (AAH85359.1) and mouse (AAH49971), and the FcRn sequences of human (NP_001129491), chimpanzee (XP_512822), macaque (AF485818_1), sheep (NP_001116875), cattle (AF221522_1), pig (NP_999362), dog (XP_533618), rabbit (NP_001116409), rat (NP_203502) and mouse (NP_034319) were downloaded. The alignments were made using Clustalω software.

The coordinates of the crystal structures of hFcRn in complex with WT HSA (PDB ID 4N0F) or an engineered HSA variant (HSA13) (PDB ID 4K71)^18,26^ were used and inspected using the PyMOL software (Schrodinger Inc.).

### Statistical analysis

Graphs were made and statistical analyses were performed using GraphPad Prism version 7.0 (GraphPad) and Microsoft Excel 2010 (Microsoft). The albumin variants were produced in two-three independent rounds. ELISA was performed at least three times and the numbers given represent the mean and standard deviation of duplicates from one representative experiment. SPR was performed two-three times and the numbers given represent the mean and standard deviation of duplicates from one representative experiment.

## Electronic supplementary material

Supplementary Information

## Data Availability

The data that support the findings of this study are available from the corresponding author upon reasonable request.
