# Supplementary material for: Human and mouse albumin bind their respective neonatal Fc receptors differently
Source: Sci Rep. 2018 Oct 2;8:14648. doi: 10.1038/s41598-018-32817-0 (PMC6168492; doi:10.1038/s41598-018-32817-0)
Supplement: Supplementary file 1 — Supplementary Information [file 41598_2018_32817_MOESM1_ESM.pdf]

SUPPLEMENTARY INFORMATION

Human and mouse albumin bind their respective neonatal Fc receptors differently

Jeannette Nilsen, Malin Bern, Kine Marita Knudsen Sand, Algirdas Grevys, Bjørn Dalhus, Inger Sandlie and Jan Terje Andersen

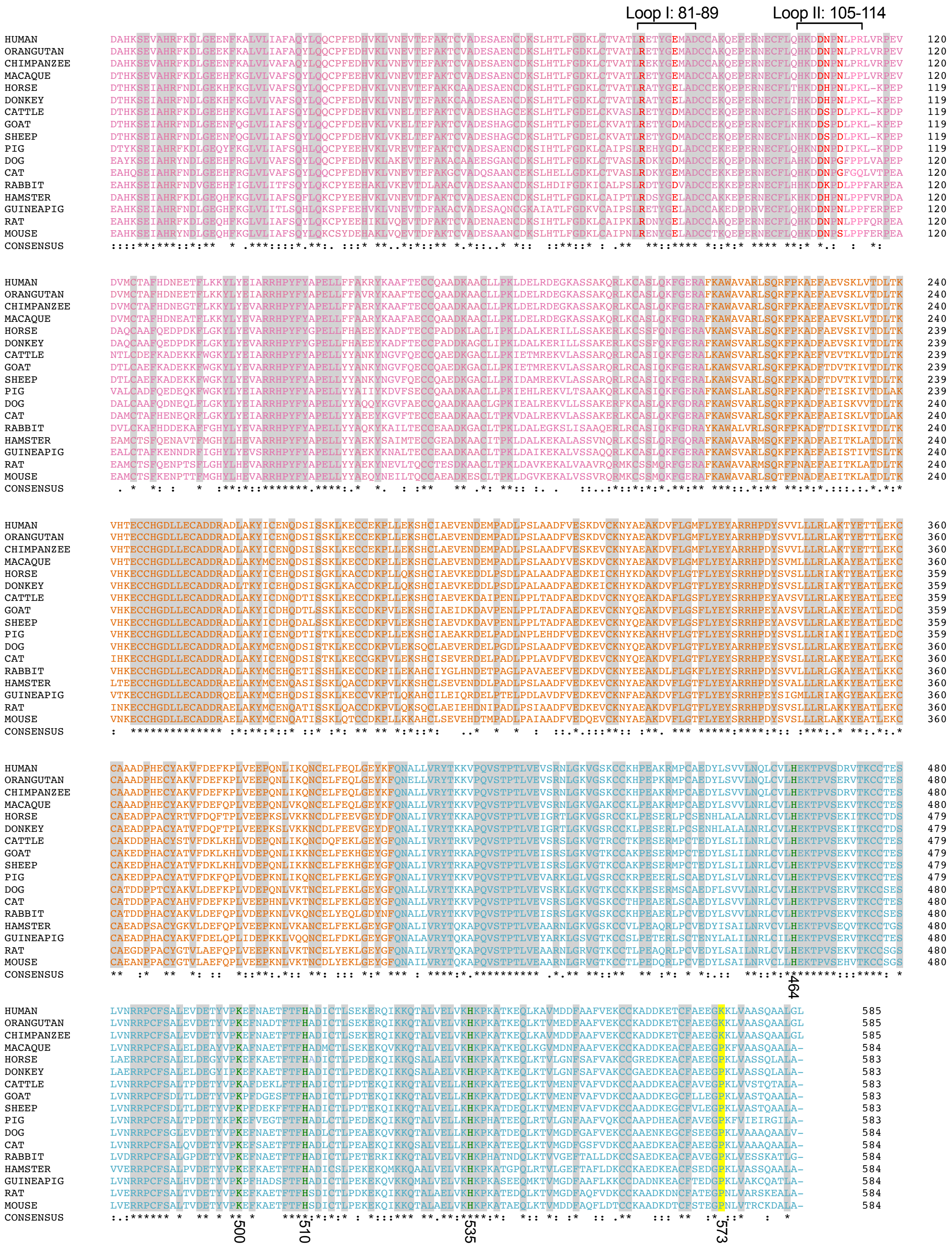

Loop I: 81-89
Loop II: 105-114
HUMAN
ORANGUTAN
CHIMPANZEE
MACAQUE
HORSE
DONKEY
CATTLE
GOAT
SHEEP
PIG
DOG
CAT
RABBIT
HAMSTER
GUINEAPIG
RAT
MOUSE
CONSENSUS
464
500
510
535
573

Supplementary Figure S1. Alignment of the amino acid sequences of albumin from multiple species. DI, DII and DIII of albumin are colored in pink, orange and blue, respectively, and amino acids that are fully conserved among species are highlighted in grey. The loops of DI involved in binding of HSA to hFcRn are indicated and loop residues that form direct interactions with the receptor in the WT HSA-hFcRn co-crystal structure are colored in red. Key residues in DIII are shown in green, while residue 573 (human numbering) is highlighted in yellow. The albumin sequences included are: human (AAA98797), orangutan (NP_001127106.2), chimpanzee (XP_517233.3), macaque (NP_001182578.1), horse (NP_001075972.1), donkey (AAV28861), cattle (AAA51411), goat (ACF10391), sheep (NP_001009376), pig (AAA30988.1), dog (CAB64867.1), cat (CAA59279.1), rabbit (NP_001075813), rat (AAH85359.1), mouse (AAH49971), hamster (ABR68005.1) and guinea pig (AAQ20088.1). The alignment was designed using the Clustalω software.

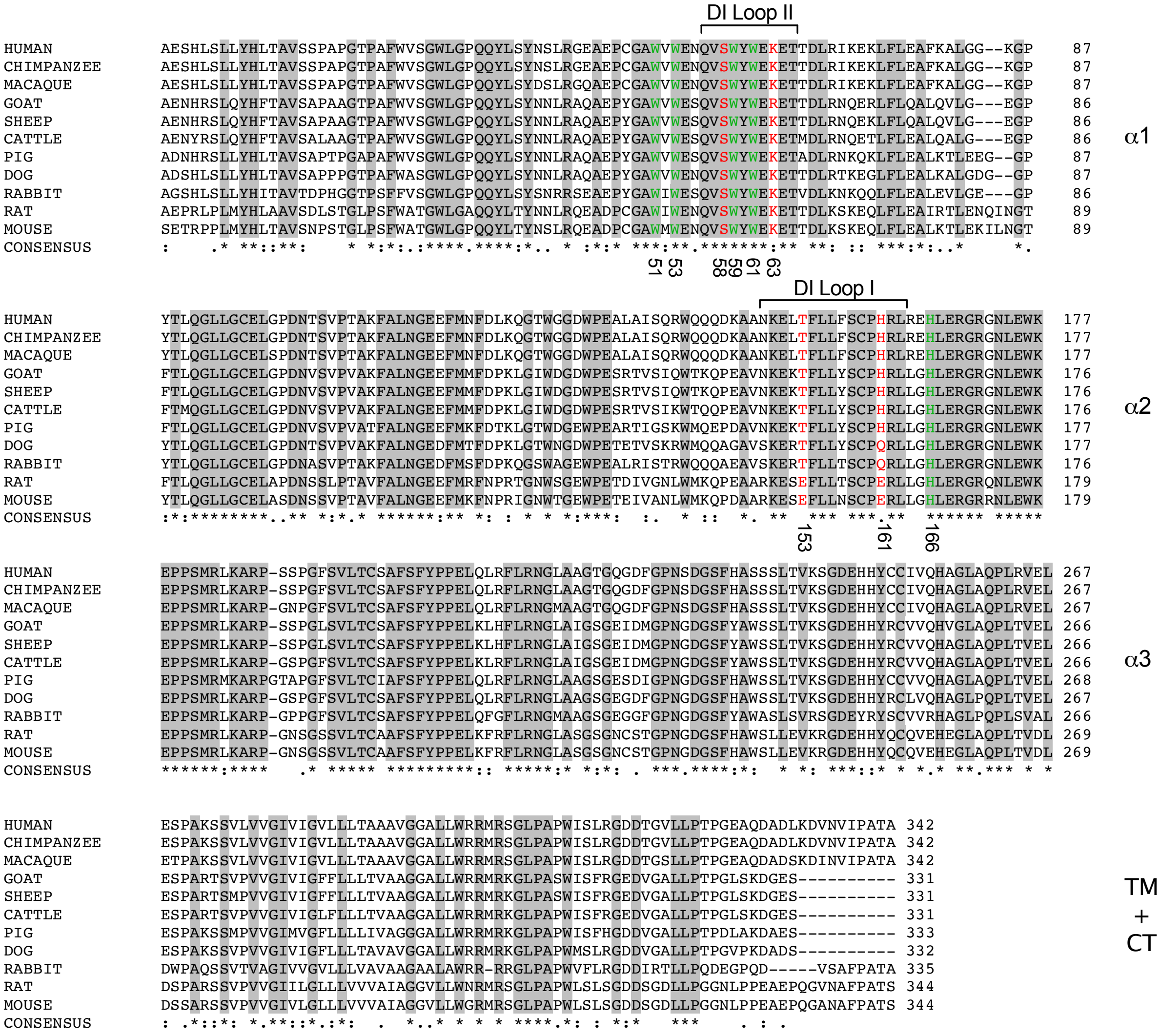

Supplementary Figure S2. Alignment of the amino acid sequences of FcRn heavy chain from multiple species. Alignment of the amino acid sequences of the heavy chains of FcRn from ten species, where the three domains (α1, α2 and α3) and the transmembrane (TM) and cytosolic tail (CT) are indicated. Amino acids that are fully conserved are highlighted in grey. Amino acid stretches that make up the interaction sites for loop I and II of albumin DI in the WT HSA-hFcRn co-crystal structure are indicated, while the residues that form direct contacts with the loops are colored in red. Key residues in albumin binding are shown in green. The FcRn sequences included are: human (NP_001129491), chimpanzee (XP_512822), macaque (AF485818_1), sheep (NP_001116875), cattle (AF221522_1), pig (NP_999362), dog (XP_533618), rabbit (NP_001116409), rat (NP_203502) and mouse (NP_034319). The alignment was designed using the Clustalω software.

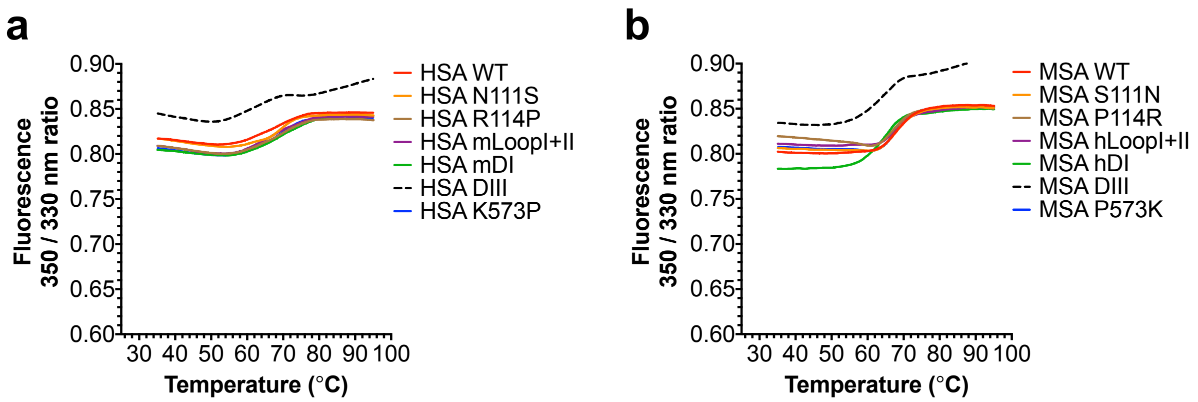

Supplementary Figure S3. Thermal unfolding curves. Representative unfolding profile curves of WT HSA and mutant variants (**a**) and WT MSA and mutant variants (**b**) obtained by intrinsic fluorescence measurements using Tycho NT. 6.

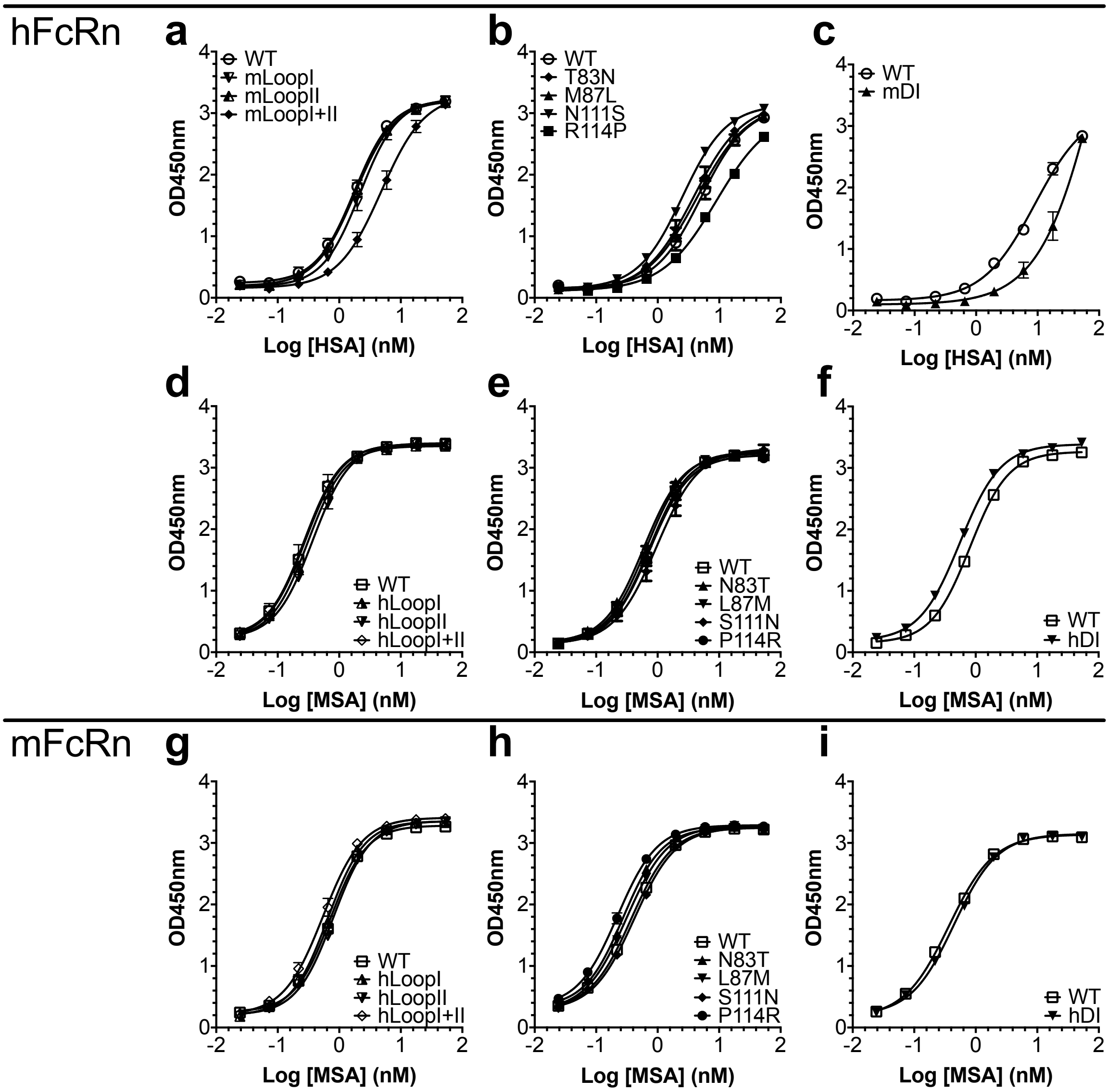

Supplementary Figure S4. Binding of engineered albumin variants to FcRn at pH 5.8. (**a-c**) ELISA showing binding of WT HSA and mutant variants to hFcRn at pH 5.8. (**d-i**) ELISA showing binding of WT MSA and mutant variants to hFcRn (**d-f**) and mFcRn (**g-i**) at pH 5.8. The curves were fitted using nonlinear regression (4 parameter). The numbers represent the mean ± s.d. of duplicates from one representative experiment.

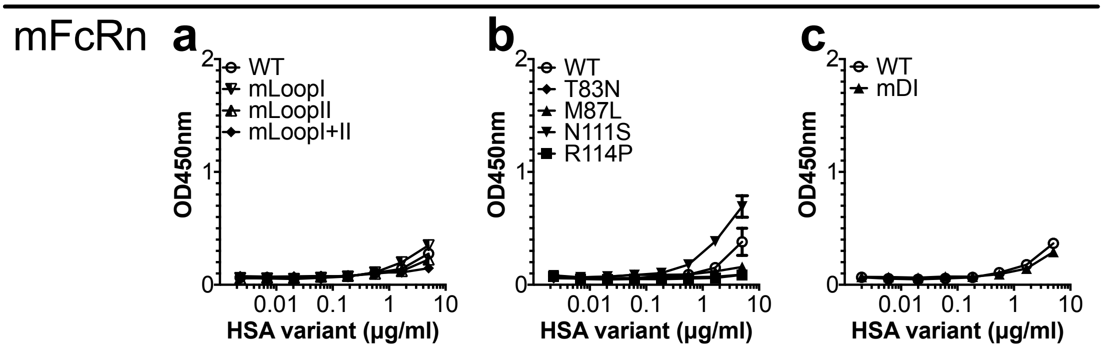

Supplementary Figure S5. HSA binds weakly to mFcRn at pH 5.8. (**a-c**) ELISA showing binding of WT HSA and engineered variants to mFcRn at pH 5.8. The numbers represent the mean ± s.d. of duplicates from one representative experiment.

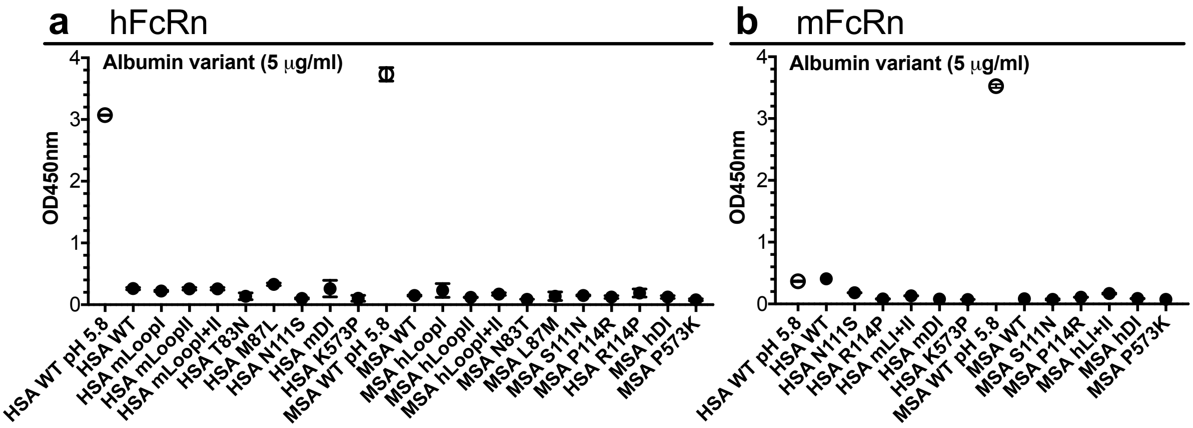

Supplementary Figure S6. No binding of albumin to FcRn at pH 7.4. ELISA showing binding of WT HSA, WT MSA and mutant variants to hFcRn (**a**) and mFcRn (**b**) at pH 7.4. Binding of WT HSA and WT MSA at pH 5.8 (indicated) was included as controls. The numbers represent the mean ± s.d. of duplicates from one representative experiment.

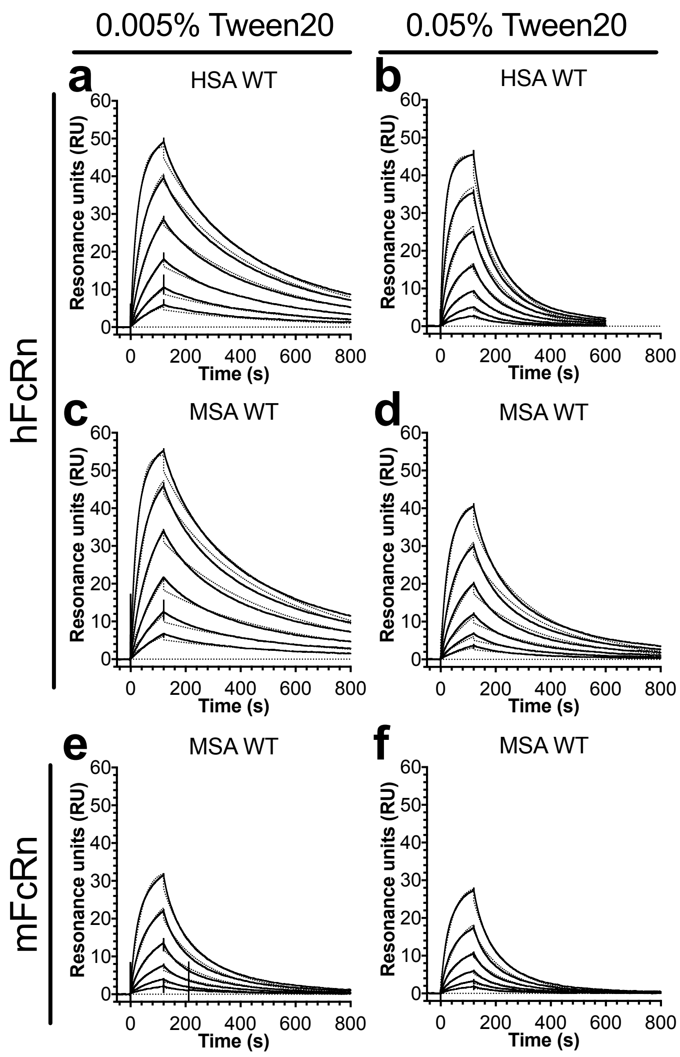

Supplementary Figure S7. Tween20 modulates binding of albumin to FcRn. Representative sensorgrams showing binding of titrated amounts of monomeric hFcRn (**a**-**d**) or mFcRn (**e**-**f**) injected over immobilized (200 RU) WT HSA (**a** and **b**) and WT MSA (**c**-**f**) in the presence of 0.005% Tween20 (**a**, **c** and **e**) or 0.05% Tween20 (**b**, **d** and **f**) at pH 5.5 (—). The fit of the data to the 1:1 binding model (·····) is shown. Injections were performed with a flow rate of 30 μl/min at 25°C.

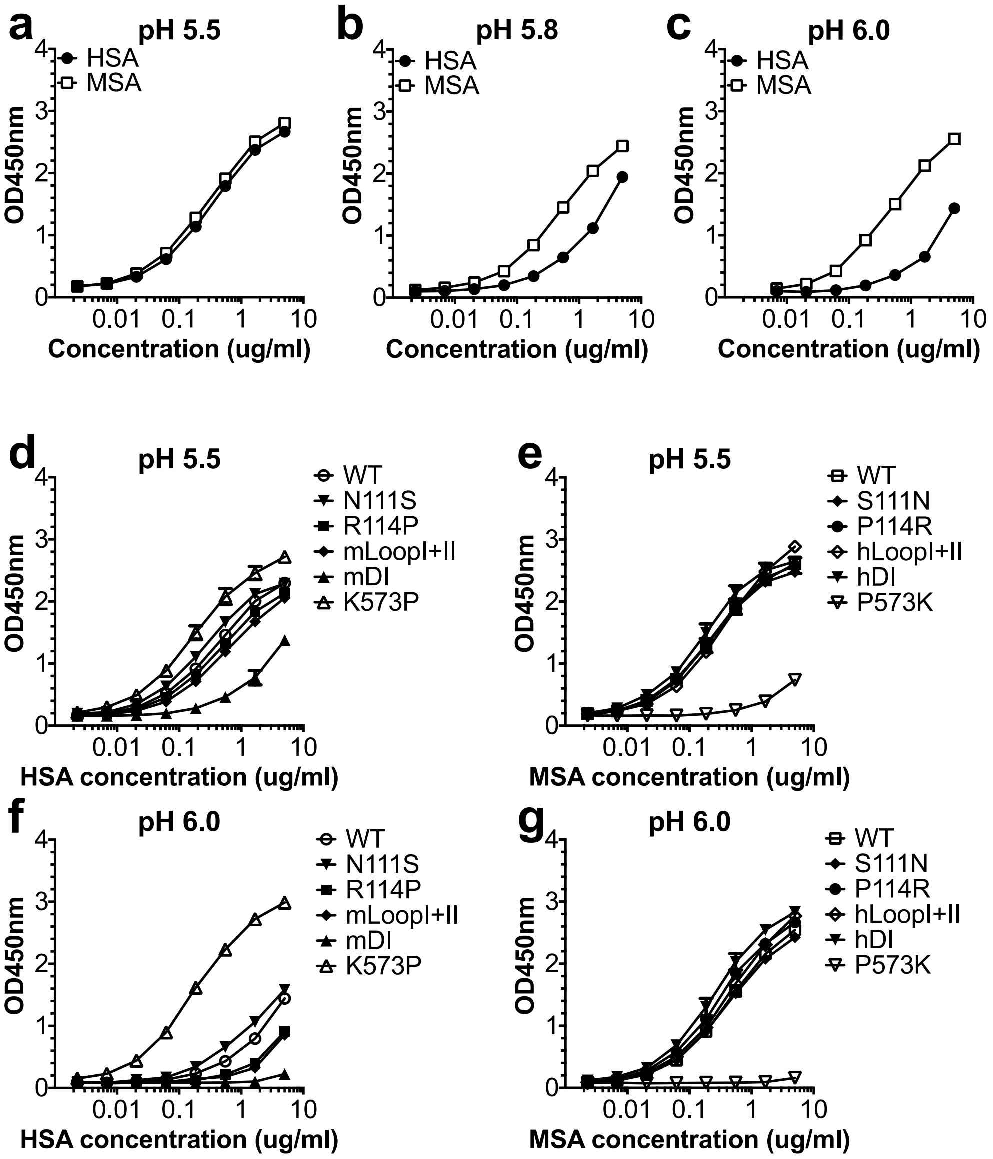

Supplementary Figure S8. The influence of pH in the 5.5-6.0 range on albumin binding to hFcRn. (**a-c**) ELISA showing binding of HSA and MSA to hFcRn at pH 5.5 (**a**), pH 5.8 (**b**) and pH 6.0 (**c**). (**d-g**) ELISA showing binding of HSA or MSA and mutant variants to hFcRn at pH 5.5 (**d** and **e**) and pH 6.0 (**f** and **g**). The numbers represent the mean ± s.d. of duplicates from one representative experiment.

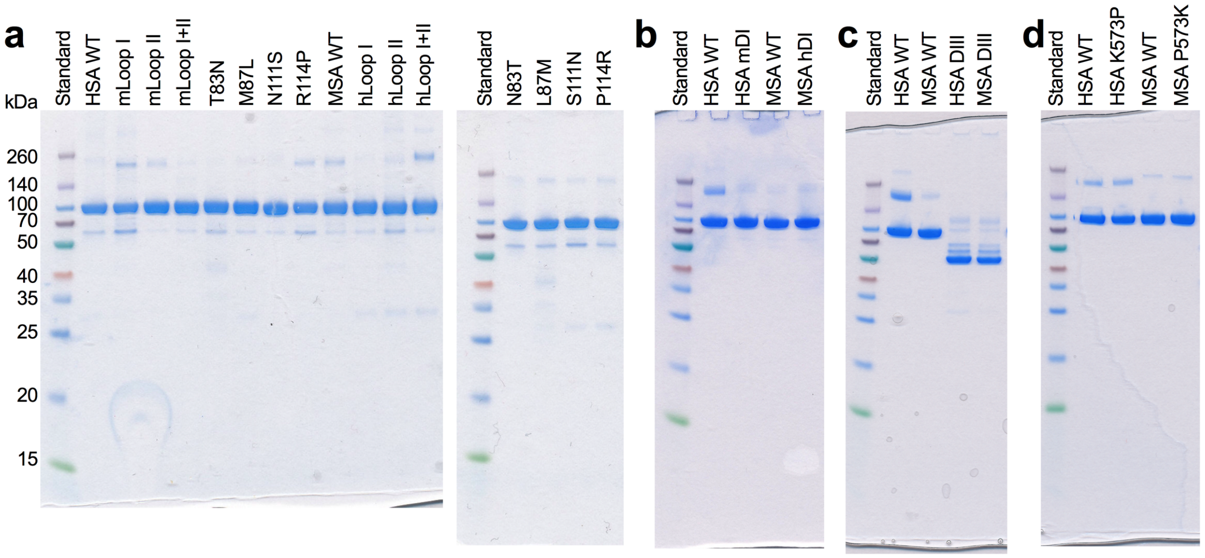

Supplementary Figure S9. Analysis of engineered albumin variants by SDS-PAGE. (a-d) 12% SDS-PAGE gels stained with Coomassie Blue showing the migration of GST-tagged HSA, MSA and mutant variants as indicated.

Supplementary Table S1. SPR-derived kinetics for binding of albumin to FcRn at two different concentrations of Tween20

VARIANT[a]	Tween20 (%)	k_a (10^4/Ms)	k_d (10^{-3}/s)	K_D[b] (nM)	χ^{2c}
HSA WT	0.005	3.8 ± 0.1	3.4 ± 0.2	89.5	0.4
hFcRn	0.05	2.3 ± 0.2	8.9 ± 0.9	387.0	0.4
MSA WT	0.005	5.1 ± 1.0	3.4 ± 0.2	66.7	0.8
hFcRn	0.05	3.2 ± 1.0	5.4 ± 1.5	168.8	0.5
MSA WT	0.005	3.8 ± 0.1	12.3 ± 0.6	323.7	0.1
mFcRn	0.05	3.0 ± 0.1	16.4 ± 2.0	546.7	0.1

[a,] The albumin variants were immobilized on CM5 chips and serial dilutions of hFcRn or mFcRn were injected.
[b,] The kinetic rate constants were obtained using a simple first-order (1:1) Langmuir bimolecular interaction model. The kinetics values represent the mean ± s.d. of duplicates.
[c,] χ^2 values resulting from curve fitting using the first-order (1:1) bimolecular interaction model. χ^2 is a measure of the average squared residual (the difference between the experimental data and the fitted curve).
